# Dual-Tracer Positron-Emission Tomography Using Prostate-Specific Membrane Antigen and Fluorodeoxyglucose for Staging of Prostate Cancer: A Systematic Review

**DOI:** 10.1155/2021/1544208

**Published:** 2021-08-18

**Authors:** Stephen McGeorge, Michael Kwok, Andrew Jiang, Louise Emmett, David A. Pattison, Paul A. Thomas, John W. Yaxley, Matthew J. Roberts

**Affiliations:** ^1^Department of Urology, Royal Brisbane and Women's Hospital, Butterfield St, Herston 4029, Queensland, Australia; ^2^Department of Urology, Redcliffe Hospital, Anzac Avenue, Redcliffe 4020, Queensland, Australia; ^3^Faculty of Medicine, The University of Queensland, Herston 4006, Queensland, Australia; ^4^Department of Theranostics and Nuclear Medicine, St. Vincent's Hospital Sydney, 390 Victoria St, Darlinghurst 2010, New South Wales, Australia; ^5^Garvan Institute of Medical Research, 384 Victoria St, Darlinghurst 2010, New South Wales, Australia; ^6^Department of Nuclear Medicine & Specialised PET Services, Royal Brisbane and Women's Hospital, Butterfield St, Herston 4029, Queensland, Australia; ^7^Wesley Urology Clinic, Wesley Hospital, 451 Coronation Dr, Auchenflower 4066, Queensland, Australia; ^8^University of Queensland Centre for Clinical Research, Faculty of Medicine, Building 71/918 RBWH, Butterfield St, Herston 4029, Queensland, Australia

## Abstract

PSMA PET is more accurate than conventional imaging (CT/bone scan) for staging of intermediate- or high-risk prostate cancer (PCa), but 5–10% of primary tumours have low PSMA ligand uptake. FDG PET has been used to further define disease extent in end-stage castrate-resistant PCa and may be beneficial earlier in the disease course for more accurate staging. The objective of this study was to review the available evidence for patients undergoing both FDG and PSMA PET for PCa staging at initial diagnosis and in recurrent disease. A systematic literature review was performed for studies with direct, intraindividual comparison of PSMA and FDG PET for staging of PCa. Assessment for radioligand therapy eligibility was not considered. Risk of bias was assessed. 543 citations were screened and assessed. 13 case reports, three retrospective studies, and one prospective study were included. FDG after PSMA PET improved the detection of metastases from 65% to 73% in high-risk early castration-resistant PCa with negative conventional imaging (M0). Positive FDG PET was found in 17% of men with negative PSMA PET for postprostatectomy biochemical recurrence. Gleason score ≥8 and higher PSA levels predicted FDG-avid metastases in BCR and primary staging. Variant histology (ductal and neuroendocrine) was common in case reports, resulting in PSMA-negative FDG-positive imaging for 3 patients. Dual-tracer PET for PCa may assist in characterising high-risk disease during primary staging and restaging. Further studies are required to determine the additive benefit of FDG PET and if the FDG-positive phenotype may indicate a poorer prognosis.

## 1. Introduction

Prostate cancer (PCa) is the most commonly diagnosed internal malignancy and high-ranking cause of mortality in men worldwide [1, 2]. An increased incidence of *de novo* metastatic PCa has been observed since 2004, most significantly in men aged 55 to 69 years, potentially contributing to higher mortality [3, 4]. Modern approaches to metastatic PCa treatment incorporate aggressive systemic therapy, such as taxane-based chemotherapy, androgen receptor inhibitors, and radioligand therapy (RLT) [5, 6]. Additionally, earlier use of systemic therapy prior to treatment of high-risk, locally advanced disease has been reported and is the subject of ongoing clinical trials [7–10]. However, accurate patient selection for systemic therapy remains one of the key challenges in urologic oncology.

Despite limitations, conventional imaging with computed tomography (CT) and technetium-based bone scan are widely used for staging [11]. However, cumulative evidence indicates that prostate-specific membrane antigen (PSMA) positron-emission tomography (PET) should be a centrepiece of staging for intermediate- to high-risk patients [11–16]. A prospective, randomised trial by Hofman and colleagues determined that PSMA PET/CT was 27% (95% CI: 23–31) more accurate than conventional imaging [12]. However, 5–10% of primary PCa tumours have low PSMA activity which evade detection by PSMA PET, mostly in high-grade and variant tumour types [14–18].

18-F-Fluorodeoxyglucose (FDG) PET use in PCa has previously been of limited benefit in primary staging and biochemical recurrence (BCR) [19, 20]. In a study of 41 men with Gleason score ≥8 PCa who had FDG PET/CT for primary staging, 11 men had nodal metastasis from histopathology, and only 3 of these men (27%) had corresponding lymph node FDG uptake [21]. Increasing use of FDG PET to aid patient selection for RLT and other therapies in mCRPCa has provided insight into tumour heterogeneity and benefits of dual-tracer PET imaging [22, 23]. Discordant findings have been attributed to the increased anaerobic glycolysis detected by FDG PET in patients with more aggressive histological types [24]. Whilst the use of PSMA and FDG PET imaging in PCa has been examined individually, the potential diagnostic impact for individual patients to undergo dual-tracer PET imaging remains unclear. Additional use of FDG PET may improve disease characterisation in patients with high-risk localised and metastatic PCa, with inconclusive conventional imaging and negative PSMA PET.

We sought to summarise the available evidence for the use of dual-tracer PET, being 18-F-fluorodeoxyglucose (FDG) PET in addition to PSMA PET, for PCa staging at initial diagnosis and in recurrent disease.

## 2. Methods

### 2.1. Search Strategy

A systematic literature search was performed in April 2021 (Table S1) in accordance with the preferred reporting items for systematic reviews and meta-analysis (PRISMA) and the Cochrane Handbook guidelines [25, 26]. The review protocol was registered (CRD42020201307) and published in the International Prospective Register of Systematic Reviews (PROSPERO). Citation searches were conducted from included reports to ensure all relevant studies were captured. All included studies received ethical approval, and the source data were publicly available, so ethical approval was not sought [27].

### 2.2. Study Selection

All original research reports that directly compared PSMA and FDG PET in the setting of histologically diagnosed or clinically suspected PCa were considered. Editorials, review articles, and animal studies were excluded, as were studies comparing PSMA and FDG PET for the purpose of RLT, as this topic was felt to be beyond the scope of the current review. Furthermore, records were excluded if they assessed other primary malignancies or active systemic disease accounting for tracer uptake, PCa was not specifically diagnosed, or they compared alternative tracers that were not FDG or PSMA.

### 2.3. Quality Assessment

Methodological quality was appraised using checklists recommended by the Cochrane Guidelines [28, 29]. Each article was assessed for bias by two independent authors, with a third reviewer used to resolve disagreements (Tables S2 and S3).

### 2.4. Data Management

The data from case reports included, where available, patient age, PSA at the time of imaging, indication for dual-tracer use, sites of metastatic lesions, ligand uptake patterns for FDG and PSMA, previous PCa treatments, histological PCa type, and Gleason score. Cases were considered to be positive if ligand uptake (according to criteria used in each case) was reported in either the prostate or at sites of metastatic disease. Discordant ligand uptake was reported when both FDG and PSMA PET were positive, but there were different sites of the disease. If multiple PSA values were reported, the PSA at the time of PET imaging was used for this comparison. For articles concerning a population, these data were collected as a range, where applicable, as well as the inclusion and exclusion criteria.

Included studies displayed significant heterogeneity and small overall population, so a quantitative assessment was not possible (Table S4).

## 3. Results

**Figure 1:**
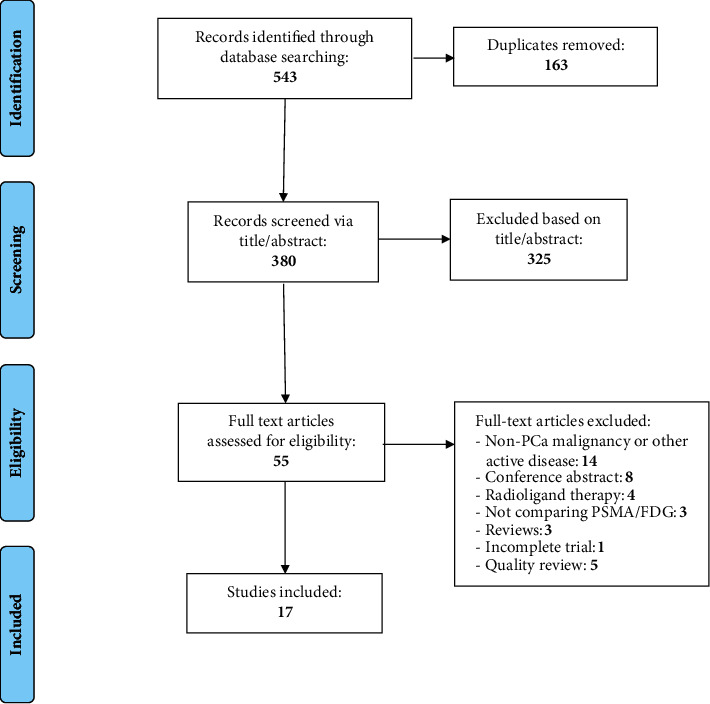
PRISMA 2009 flow diagram.

The database search yielded 543 citations. Following screening and assessment (Figure 1), one single-arm prospective trial, three retrospective observational studies, and 13 case reports were included in the final analysis (Tables 1 and 2).

### 3.1. Castration-Resistant Disease

A prospective single-arm trial investigated the prevalence of PSMA-negative FDG-positive lesions in 37 men with negative conventional imaging (M0) in the setting of high-risk early castration resistance (defined as PSA progression with PSA ≤2 ng/ml and PSA doubling time ≤10 months) who were restaged using PSMA and then FDG PET [33]. 65% of men had metastatic disease on initial PSMA PET, while an additional 5% had localised PSMA avidity within the prostate, which was concordant with FDG PET. Among 30% of men with no PSMA avidity, 27% (8% overall) showed metastatic disease according to FDG PET, increasing the detection of metastases from 65% to 73%.

High-grade disease, defined as Gleason grade group of four or higher, correlated with nodal or distant metastases on FDG PET (OR = 13.09, *p*=0.02). A PSA doubling time less than six months was associated with the presence of PSMA-positive metastatic disease (OR = 8.00, *p*=0.009), but not for FDG-positive metastases (*p*=0.29). Discordant lesions (PSMA-negative FDG-positive) were more common for axial bone lesions compared to concordant PSMA-FDG-positive patients (*p*=0.02). Metastatic disease burden was according to CHAARTED criteria and was unchanged in the use of FDG PET [47]. However, use of FDG PET was estimated to enable an additional six men (19 in total) precise lesion targeting for oligometastatic-directed therapy according to SABR-COMET trial criteria [48].

Within the same manuscript, an additional analysis of a retrospective cohort of 41 men was reported. The cohort included 14 men with BCR, 18 men with hormone-sensitive PCa (HSPCa), and 9 men with mCRPCa. Metastatic disease according to PSMA PET was detected in 29%, 39%, and 100% of men (with FDG concordant disease for 100%, 86%, and 67%) with BCR, HSPCa, and mCRPCa, respectively. PSMA-negative FDG-positive disease was rare in HSPCa (6%) and more commonly observed for CRPCa (33%).

### 3.2. Biochemically Recurrent Hormone-Sensitive Disease

Predictive factors for FDG-positive PET in men with BCR (PSA >0.2 ng/ml) after radical prostatectomy and a negative PSMA PET were determined in a retrospective case-control study (*n* = 72) [32]. The median PSA was 3.4 ng/ml, and 53% had prior adjuvant therapy. FDG PET was positive in 17% of men, where serum PSA (16.7 versus 0.8 ng/ml) and Gleason scores (34.4% with Gleason score ≥8 vs. 2.5% with Gleason score <8; *p* < 0.001) were higher than the FDG-negative group. There were no differences in age, PSA doubling time, interval between PSMA and FDG PET, or adjuvant therapies. A PSA cutoff of 2.3 ng/ml was 91.7% accurate for FDG PET positivity (area under the curve of 0.872, *p* < 0.001). The group was divided into three groups for the prediction of positive FDG in the setting of negative PSMA, being low potential (PSA <2.3 ng/ml and Gleason score <8), moderate potential (PSA ≥2.3 ng/ml or Gleason score ≥8, but not both), and high potential (PSA ≥2.3 ng/ml and Gleason score ≥8). These groups showed 0% (36/36), 11.5% (3/26), and 90% (9/10) positive FDG PET results, respectively.

A retrospective evaluation of 138 men who underwent ^68^Ga-PSMA-11 and FDG PET for differentiating lymph node metastases from peripheral ganglia was examined for oncological outcomes [31]. Imaging was for primary staging in 47% (*n* = 65) and BCR in 53% (*n* = 73). 47% of men had Gleason ≥8 disease, and mean PSA was 56.4 ng/mL (IQR: 18.5–99.7) and 1.1 ng/mL (IQR: 0.4–4.1) in the primary staging and BCR cohorts, respectively. 42% of patients demonstrated PSMA-avid lymph node metastases, totalling 83 metastases, with FDG concordance in 63% (*n* = 52). Significantly less ganglia showed FDG uptake (*p* < 0.001), so the PSMA/FDG PET combination was shown to detect lymph node metastases when both were avid (PSMA SUV_max_ >2.05 and FDG SUV_max_ >4.1; 43/47, 92%) compared to low or no avidity (PSMA SUV_max_ <2.05 and FDG SUV_max_ <4.1; 3/334, 1%). No correlation was found for PSMA or FDG uptake (according to SUV_max_) with Gleason scores or PSA level (binary grouping, not defined, *p* > 0.05) in both primary staging and BCR cohorts.

### 3.3. Primary Staging

Staging with dual-tracer PSMA/FDG PET/CT prior to treatment was examined in one retrospective study of 21 men [30]. 68.7% (*n* = 11/16) of men had Gleason scores ≥8, and median PSA at diagnosis was 41.2 ng/mL (range: 5–200). All patients had ^18^F-PSMA-1007 uptake within the prostate; however, 66.7% had local FDG uptake. PSMA PET/CT identified more bone (50 vs. 32) and lymph node (25 vs. 22) metastases, with less benign lesions (21% vs. 49%) than FDG PET. Most patients with metastases on FDG PET had Gleason scores ≥8. SUV_max_ (median: 10.72 vs. 4.42), SUV_mean_ (median: 6.67 vs. 2.59), and tumour-to-background ratio (13.3 vs. 7.91) of identified metastases were all higher for ^18^F-PSMA-1007 than FDG PET (*p* < 0.001).

### 3.4. Case Reports

Case report data are summarised in Table 2 (Ref. A–C) according to indication for dual-tracer use with subgrouping according to the ligand uptake pattern. The most common indication was for men with BCR requiring characterisation of lung nodules seen on conventional imaging (5/14 patients) [34, 39, 41–43]. Variant histology was also common, with three cases of ductal variant and three cases of neuroendocrine differentiation, although one of these was presumed on the basis of a raised serum chromogranin A level in combination with DOTATOC PET avidity [34, 37–40].

PSMA and FDG ligand uptake patterns were discordant in the majority of cases, although this was not consistently reported for each individual lesion in every patient (Table 2). Three cases reported a negative PSMA PET with positive FDG PET, all of which had either ductal or neuroendocrine histology. Serum PSA levels were highly variable for all patterns of ligand uptake according to the tracer, being 0.2–37 ng/ml for discordant PSMA-positive FDG-positive, 0.2–49 ng/ml for PSMA-positive FDG-negative, and 0.59–163 ng/ml in PSMA-negative FDG-positive.

## 4. Discussion

The role of dual-tracer PET for localised or recurrent PCa is unclear, and available data are limited. Despite significant heterogeneity in patient populations, indications, and performance which limit meta-analysis, discordant imaging and altered staging classification were observed. Imaging discordance was more likely to be observed in more advanced disease, presumably due to greater tumour clone heterogeneity.

An important application of dual-tracer PET/CT may be in the identification of additional sites of disease amenable to oligometastatic directed therapy. In the early castrate-resistant population discussed by Wang et al., 24% of patients had at least one PSMA− FDG+ lesion which would have been otherwise missed [33]. The SABR-COMET trial found an overall survival benefit with stereotactic ablative radiotherapy, and results from the ORIOLE trial showed that the total consolidation of all PSMA-avid diseases gave significant progression-free survival and distant metastasis-free survival advantages [48, 49]. Therefore, dual PSMA/FDG PET/CT may improve oncological outcomes by identifying additional sites of the disease amenable to oligometastatic directed therapy.

Furthermore, the PSMA-negative FDG-positive phenotype may represent a population at a greater risk of rapid disease progression. These poor oncological outcomes have been reported in the context of RLT, with Suman and colleagues reporting that, among 40 patients who underwent ^177^Lu-PSMA-617 RLT, high FDG uptake (SUV_max_ >15) was reported to correlate with worse 12-month progression-free survival (PFS; *p*=0.05) and poor response to RLT (disease progression in 12/15 patients) [50]. Furthermore, a study of 16 men with poor PSMA avidity or significantly discordant FDG avidity ineligible for ^177^Lu-PSMA-617 RLT reported a median overall survival of 2.3 months when FDG avidity was high [51]. While PSMA-negative imaging may suggest aggressive disease, a lower ratio of PSMA SUV_max_ to FDG SUV_max_ may better predict PSA reduction ≥30% following RLT (*p* < 0.02) [23]. However, FDG findings alone were not predictive of response, and no imaging factors correlated with PSA response ≥50%. Conversely, 59% of men with low FDG uptake (SUV_max_ <15) reported improved or stable disease with RLT in one study, while absent PSMA and FDG avidity following RLT was indicative of favourable treatment response [50].

We observed low PSMA avidity in FDG-positive disease to be a common finding among papers included in this review. Low PSMA avidity in advanced PCa is hypothesised to be due to dedifferentiation or neuroendocrine transformation. Biomarker-focused research supports this hypothesis, with a retrospective analysis in 66 patients referred for RLT, of which 41 patients (62%) had at least one PSMA-negative FDG-positive lesion and demonstrated higher levels of neuron-specific enolase, a neuroendocrine marker [52]. Furthermore, neuroendocrine PCa has a more aggressive course and thereby an expectation of increased anaerobic glycolysis with consequent FDG avidity [24]. This is in keeping with existing evidence on divergent clonal evolution of CRPCa and the “genomic overlap” between conventional adenocarcinoma and neuroendocrine PCa in the development of castration resistance [53]. There is also support in the correlation between levels of glucose uptake-associated genes with neuroendocrine gene signatures and low PSMA expression [54]. Given the poor prognosis in these patients, further research on dual-tracer PET may help streamline earlier diagnosis of this PCa phenotype and thereby prompt earlier escalation to more aggressive treatment to improve oncological outcomes.

PSMA PET has been shown to be superior to conventional imaging for the initial staging of intermediate- and high-risk PCa [12]. However, approximately 5–10% of primary PCa has insufficient PSMA avidity for PET detection due to contributions of both low- (usually of lower volume) and high-grade disease [14]. In the latter population, the available data suggest that FDG may detect primary and metastatic disease that would otherwise not be observed on PSMA PET or conventional imaging. Thus, the potential for FDG PET use in the primary staging of high-grade PCa, especially with neuroendocrine or ductal variation, may be an early independent identifier of aggressive disease and poorer oncological outcomes with conventional therapies.

The limitations of this review include limited available studies and details of data included in these studies, including their mostly retrospective nature and limited coverage of each disease state. Furthermore, we were unable to adjust for selection and publication bias in the included studies. Conversely, to our knowledge, this review represents the first summary of the available literature of the intraindividual assessment of PSMA and FDG PET.

In conclusion, the diagnostic utility of dual-tracer FDG/PSMA PET/CT for PCa may assist in characterising high-risk disease during primary staging and restaging. Addition of FDG PET can identify additional sites of the disease amenable to oligometastatic directed therapy. Patients with FDG-positive disease in advanced PCa states have a poor prognosis, especially with concurrently negative PSMA PET. When applied to high-risk (high-grade or variant histology), hormone-sensitive disease in the localised or recurrent stage, detection of the FDG-positive phenotype may signal a poorer prognosis to prompt more aggressive intervention earlier in the disease course. Further studies are required to validate the prognostic insights outlined here and selective incorporation into therapeutic clinical trials.

## Figures and Tables

**Table 1:** Summary of included cohort studies, comprising prospective trials and retrospective analyses, across the spectrum of prostate cancer (PCa) including primary staging, biochemical recurrence (BCR), hormone-sensitive PCa (HSPCa), and castrate-resistant PCa (CRPCa).

Category	*N*	Study	*N*	Criteria	Characteristics	Findings
Primary staging	86	Zhou et al. [30]	21	(i) No prior treatment	Median PSA: 41.2 ng/mL 53% ISUP ≥4	PSMA PET/CT identified more bone (50 vs. 32) and lymph node (25 vs. 22) metastases, with less benign lesions (21% vs. 49%) than FDG PET
		Shi et al. [31]	65	(i) No treatment between PSMA and FDG PET/CT (ii) <2 weeks of interval in imaging	Median PSA: 56.4 ng/ml	PSMA/FDG PET detected N+ when both are avid (PSMA SUV_max_ >2.05 and FDG SUV_max_ >4.1; *n* = 43/47, 92%) compared to low or no avidity (PSMA SUV_max_ <2.05 and FDG SUV_max_ <4.1; *n* = 3/334, 1%) 83 PSMA-avid lymph nodes (primary + BCR) with FDG uptake in 53% (*n* = 52/83) No association between the PSA level and PSMA or FDG uptake in ganglia or lymph node metastases in primary staging

BCR	159	Chen et al. [32]	72	(i) PSA >0.2 ng/ml after RP (ii) Not on androgen deprivation (iii) N0M0 on Ga68 PSMA PET/CT	Median PSA: 0.5 ng/mL 44% ISUP ≥4 38% prior adjuvant ADT	FDG-avid disease found in 17% of patients with negative PSMA PET/CT
		Shi et al. [31]	73	(i) No treatment between PSMA and FDG PET/CT (ii) <2 weeks of interval in imaging	Median PSA: 1.1 ng/mL	PSMA/FDG PET detected N+ when both are avid (PSMA SUV_max_ >2.05 and FDG SUV_max_ >4.1; *n* = 43/47, 92%) compared to low or no avidity (PSMA SUV_max_ <2.05 and FDG SUV_max_ <4.1; *n* = 3/334, 1%) 83 PSMA-avid lymph nodes (primary + BCR) with FDG uptake in 53% (*n* = 52/83) No association between the PSA level and PSMA or FDG uptake in ganglia or lymph node metastases in BCR
		Wang et al. [33] (retrospective)	14	(i) Not defined	Median PSA: 0.35 ng/mL 50% ISUP ≥4 100% after RP	0% with PSMA− FDG+ lesions

HSPCa	18	Wang et al. [33] (retrospective)	18	(i) Not defined	Median PSA: 14.5 ng/mL 33% on ADT	29% of patients N+/M+ on PSMA PET/CT Detection of at least one PSMA− FDG+ lesion in 6%

CRPCa	46	Wang et al. [33] (trial group)	37	(i) Rising PSA ≤2 ng/ml (ii) Testosterone <50 ng/dL (iii) PSA doubling time ≤10 months (iv) N0M0 on CT and bone scan	Median PSA: 0.57 ng/mL 73% ISUP ≥4 100% after RP 19% postop EBRT	Subsequent addition of FDG after PSMA PET/CT increased detection of N+/M+ from 65% to 73% Detection of at least one PSMA− FDG+ lesion in 24%
		Wang et al. [33] (retrospective)	9	(i) Not defined	Median PSA: 2.26 ng/mL 89% ISUP ≥4	Detection of at least one PSMA− FDG+ lesion in 33%

**Table 2:** Summary of case report data, considering use at different stages of prostate cancer (PCa) including primary staging, recurrent disease, and castrate-resistant PCa.

Ref.	Reason for dual-tracer PET	Avidity pattern	Metastatic/localised	Histology	PSMA+ lesions	FDG+ lesions	PSA	Gleason score
A-primary staging								
[34]	Staging for ductal variant	PSMA+ FDG+ discordant	L	Ductal	Prostate, mild bilaterally	Prostate, extensively in the left lobe	0.2	4 + 5 = 9
[35]	Synchronous rectal cancer and PCa	PSMA+ FDG+ discordant	L	Adenocarcinoma	Prostate	Rectum, presacral lymph node	37	Not documented
[36]	High-grade histology, low PSMA avidity of the prostate, equivocal staging	PSMA+ FDG+ discordant	M	Adenocarcinoma	Pelvic lymph nodes, T8 vertebrae	Prostate, pelvic lymph nodes, T8 vertebrae, right acetabulum	16.3	5 + 5 = 10
[37]	Infection vs. locally advanced disease in frail elderly patient	PSMA+ FDG−	M	Neuroendocrine (presumed)	Prostate, bladder	None	49	No histology
[38]	Staging for neuroendocrine PCa	PSMA− FDG+	M	Neuroendocrine	None	Prostate, bladder, rectum, lymph nodes, bone	163	5 + 4 = 9

B-restaging of the recurrent disease								
[34]	BCR in ductal variant with lung nodules	PSMA− FDG+	M	Ductal	None	Lung	5.9	4 + 5 = 9
[39]	BCR with lung nodules	PSMA− FDG+	M	Ductal	None	Lung	0.59	Not documented
[40]	Neuroendocrine transformation	PSMA+ FDG+ discordant	M	Neuroendocrine	Bone	Bone, left pelvic soft tissue, penis	8	4 + 3 = 7
[41]	BCR with lung nodules	PSMA+ FDG+ discordant	M	Adenocarcinoma	Two lung nodules	One lung nodule	0.275	Not documented
[42]	BCR with lung nodules	PSMA+ FDG+ discordant	M	Adenocarcinoma Sarcomatoid metastasis	Pelvic lymph node, lung	Lung	4.63	4 + 4 = 8
[43]	BCR with lung nodules	PSMA+ FDG−	M	Adenocarcinoma Mucinous metastasis	Lung	None	0.2	4 + 3 = 7
[44]	BCR with negative FDG PET	PSMA+ FDG−	M	Adenocarcinoma	Mediastinal lymph nodes	None	7.1	4 + 4 = 8

C-castrate-resistant prostate cancer								
[45]	Onset of castration resistance with unclear FDG PET	PSMA+ FDG+ discordant	M	Not documented	Prostate, bone, pelvic lymph nodes	Prostate, mild bone avidity	10	Not documented
[46]	Onset of castration resistance with adrenal mass	PSMA+ FDG+ concordant	M	Adenocarcinoma	Right adrenal mass	Right adrenal mass	1.9	4 + 5 = 9

## Data Availability

The search strategies for each database used for the systematic review are included in the supplementary data. The PRISMA flowchart shows inclusion/exclusion of citations. Citations excluded in screening are held in a Covidence database by the authors and can be made available if required. Articles excluded by quality review are listed in the supplementary materials [55–59].

## Supplementary Materials

Supplementary Materials Table S1: search strategy by the database. Table S2: risk of bias assessment using QUADAS-2. Table S3: risk of bias assessment for case reports, Joanna Briggs Institute Checklist for Case Reports. Table S4: summary of articles.
