# Supplementary material for: Dual-Tracer Positron-Emission Tomography Using Prostate-Specific Membrane Antigen and Fluorodeoxyglucose for Staging of Prostate Cancer: A Systematic Review
Source: Adv Urol. 2021 Aug 18;2021:1544208. doi: 10.1155/2021/1544208 (PMC8387192; doi:10.1155/2021/1544208)
Supplement: Supplementary Materials — Table S1: search strategy by the database. Table S2: risk of bias assessment using QUADAS-2. Table S3: risk of bias assessment for case reports, Joanna Briggs Institute Checklist for Case Reports. Table S4: summary of articles. [file 1544208.f1.docx]

**Supplementary Table 1** - Search strategy by database

**MEDLINE**

| **No.** | **Query** | **Results** |
| --- | --- | --- |
| 1 | prostat* AND "prostate cancer"[tiab] | 120,638 |
| 2 | (68Ga OR "Gallium Radioisotopes"[Mesh] OR PSMA OR "prostate specific membrane antigen") AND (PSMA[tiab] OR "prostate specific membrane antigen"[tiab]) | 4,087 |
| 3 | (FDG OR fluorodeoxyglucose OR fludeoxyglucose) AND (FDG[tiab] OR fluoro-2-deoxyglucose[tiab] OR fluorodeoxyglucose[tiab] OR fludeoxyglucose[tiab]) | 41,900 |
| 4 | ("positron emission tomography" OR PET) AND ("positron emission tomography"[tiab] OR "PET"[tiab]) | 123,384 |
| **5** | **(((prostat* AND "prostate cancer"[tiab]) AND ((68Ga OR "Gallium Radioisotopes"[Mesh] OR PSMA OR "prostate specific membrane antigen") AND (PSMA[tiab] OR "prostate specific membrane antigen"[tiab]))) AND ((FDG OR fluorodeoxyglucose OR fludeoxyglucose) AND (FDG[tiab] OR fluoro-2-deoxyglucose[tiab] OR fluorodeoxyglucose[tiab] OR fludeoxyglucose[tiab]))) AND (("positron emission tomography" OR PET) AND ("positron emission tomography"[tiab] OR "PET"[tiab]))** | **112** |

**Embase**

| **No.** | **Query** | **Results** |
| --- | --- | --- |
| 1 | ('gallium 68'/exp OR 'gallium 68' OR '68ga'/exp OR 68ga OR 'gallium'/exp OR gallium OR 'prostate specific membrane antigen'/exp OR 'prostate specific membrane antigen') AND (psma:ti,ab OR 'prostate specific membrane antigen':ti,ab) AND [english]/lim | 6446 |
| 2 | 'fluorodeoxyglucose f 18'/exp AND (fdg OR fluorodeoxyglucose OR fludeoxyglucose OR 'fluoro 2 deoxyglucose') AND (fdg:ti,ab OR fluorodeoxyglucose:ti,ab OR fludeoxyglucose:ti,ab OR 'fluoro 2 deoxyglucose':ti,ab) AND [english]/lim | 40,301 |
| 3 | ('prostate cancer'/exp OR 'prostate tumor'/exp OR 'prostate cancer*' OR 'prostate tumor*') AND prostat*:ti,ab AND [english]/lim | 215,338 |
| 4 | **('prostate cancer'/exp OR 'prostate tumor'/exp OR 'prostate cancer*' OR 'prostate tumor*') AND prostat*:ti,ab AND ('fluorodeoxyglucose f 18'/exp OR fdg OR fluorodeoxyglucose OR fludeoxyglucose OR 'fluoro 2 deoxyglucose') AND (fdg:ti,ab OR fluorodeoxyglucose:ti,ab OR fludeoxyglucose:ti,ab OR 'fluoro 2 deoxyglucose':ti,ab) AND ('gallium 68'/exp OR 'gallium 68' OR '68ga'/exp OR 68ga OR 'gallium'/exp OR gallium OR 'prostate specific membrane antigen'/exp OR 'prostate specific membrane antigen') AND (psma:ti,ab OR 'prostate specific membrane antigen':ti,ab) AND [english]/lim** | 225 |

**Cochrane Library**

| **No.** | **Query** | **Results** |
| --- | --- | --- |
| 1 | MeSH descriptor: [Fluorodeoxyglucose F18] explode all trees | 600 |
| 2 | MeSH descriptor: [Positron-Emission Tomography] explode all trees | 1020 |
| 3 | ("prostate specific membrane antigen" OR "psma" OR "68 Ga" OR "68Ga" OR "gallium"):ti,ab,kw | 730 |
| 4 | ("FDG" OR "fluorodeoxyglucose" OR “fludeoxyglucose” OR “fluoro 2 deoxyglucose”):ti,ab,kw | 2438 |
| 5 | (prostat*):ti,ab,kw | 22,327 |
| 6 | ("positron emission tomography" OR "PET"):ti,ab,kw | 8123 |
| 7 | #2 AND #6 | 1020 |
| 8 | #1 AND #4 | 600 |
| **9** | **#3 AND #4 AND #5 AND #6** | **14** |

**Web of Science**

| **No.** | **Query** | **Results** |
| --- | --- | --- |
| 1 | TS=(prostat*) AND LANGUAGE:(English) | 326,417 |
| 2 | TS=(FDG OR fluorodeoxyglucose OR fludeoxyglucose OR ‘fluoro-2-deoxyglucose’) AND LANGUAGE: (English) | 62,137 |
| 3 | TS=(PSMA OR 'prostate specific membrane antigen') AND LANGUAGE: (English) | 7165 |
| 4 | TS=('positron emission tomography' OR PET) AND LANGUAGE: (English) | 239,515 |
| 5 | #4 AND #3 AND #2 AND #1 | 209 |
| **6** | **(#4 AND #3 AND #2 AND #1 AND (AB=(PSMA OR 'prostate specific membrane antigen' OR FDG OR fluorodeoxyglucose OR fludeoxyglucose OR '‘fluoro-2-deoxyglucose') OR TI=(PSMA OR 'prostate specific membrane antigen' OR FDG OR fluorodeoxyglucose OR fludeoxyglucose OR 'fluoro-2-deoxyglucose'))) AND LANGUAGE: (English)** | **199** |

**Supplementary Table 2** – Risk of bias assessment, QUADAS-2 [29]

|  | **Wang et al [30]** | | **Chen et al [33]** | | **Shi et al [34]** | | **Zhou et al [35]** | |
| --- | --- | --- | --- | --- | --- | --- | --- | --- |
|  | **Review 1** | **Review 2** | **Review 1** | **Review 2** | **Review 1** | **Review 2** | **Review 1** | **Review 2** |
| Was a consecutive or random sample of patients enrolled? | Yes | Yes | Yes | Yes | Yes | Yes | Unclear | Unclear |
| Was a case-control design avoided? | Yes | Yes | No | No | Yes | Yes | Yes | Yes |
| Did the study avoid inappropriate exclusions? | Yes | Yes | No | No | Yes | Yes | Yes | Unclear |
| Were the index test results interpreted without knowledge of the results of the reference standard? | Unclear | Yes | Unclear | Unclear | Unclear | Unclear | Unclear | Unclear |
| If a threshold was used, was it pre-specified? | No | No | Yes | Yes | No | No | Yes | Yes |
| Is the reference standard likely to correctly classify the target condition? | Yes | Yes | Yes | Yes | Unclear | Unclear | Yes | Yes |
| Were the reference standard results interpreted without knowledge of the results of the index test? | Unclear | Unclear | Unclear | Unclear | Unclear | Unclear | Unclear | Unclear |
| Was there an appropriate interval between index test(s) and reference standard? | Yes | Yes | Yes | Yes | Yes | Yes | Yes | Yes |
| Did all patients receive a reference standard? | Yes | Yes | Yes | Yes | Yes | Yes | Yes | Yes |
| Did patients receive the same reference standard? | Yes | Yes | Yes | Yes | Yes | Yes | Yes | Yes |
| Were all patients included in the analysis? | Yes | Yes | No | Yes | Yes | Yes | Yes | Yes |
| **Overall appraisal** | **Include** | **Include** | **Include** | **Include** | **Include** | **Include** | **Include** | **Include** |

**Supplementary Table 3** – Risk of bias assessment, Joanna Briggs Institute Checklist for Case Reports [28]

|  | **Kichloo et al[50]** | **Polverari et al[38]** | **Perez et al[36]** | **Parghane et al(41)** | **Cakir et al[51]** | **Acar et al[54]** | **Sarikaya et al[53]** | **Parida et al[42]** | **Prati et al[56]** | **McEwan et al[39]** | **Shetty et al[40]** | **Queiroz et al[52]** | **Geraldo et al[37]** | **Chen et al[43]** | **Cornelis et al[57]** | **Liu et al[58]** | **Soydal et al[59]** | **McGeorge et al[55]** |
| --- | --- | --- | --- | --- | --- | --- | --- | --- | --- | --- | --- | --- | --- | --- | --- | --- | --- | --- |
| **Reviewer 1** |  |  |  |  |  |  |  |  |  |  |  |  |  |  |  |  |  |  |
| Were patient’s demographic characteristics clearly described? | Yes | Yes | Yes | Yes | Yes | Yes | Yes | Yes | Yes | Yes | Yes | Yes | Yes | Yes |  |  |  |  |
| Was the patient’s history clearly described and presented as a timeline? | Yes | Yes | Yes | Yes | Yes | No | Yes | Yes | Yes | Yes | Yes | Yes | Yes | No |  |  |  |  |
| Was the current clinical condition of the patient on presentation clearly described? | Yes | Yes | No | Yes | Yes | No | Yes | Unclear | Yes | Yes | Yes | Yes | Yes | Yes |  |  |  |  |
| Were diagnostic tests or assessment methods and the results clearly described? | Yes | Yes | Yes | Yes | Yes | Yes | Yes | Yes | No | Yes | Yes | Yes | Yes | Yes |  |  |  |  |
| Was the intervention(s) or treatment procedure(s) clearly described? | Yes | Yes | Yes | Yes | Yes | Yes | Yes | Yes | Yes | Yes | Yes | Yes | Yes | Yes |  |  |  |  |
| Was the post-intervention clinical condition clearly described? | Yes | Yes | Yes | Yes | No | No | Yes | No | Unclear | No | Yes | Unclear | Yes | No |  |  |  |  |
| Were adverse events (harms) or unanticipated events identified and described? | No | No | No | No | No | No | No | No | No | No | No | No | No | No |  |  |  |  |
| Does the case report provide takeaway lessons? | Yes | Yes | Unclear | Yes | Yes | No | Yes | Yes | Yes | Yes | Yes | No | Yes | Unclear |  |  |  |  |
| **Overall appraisal** | **Include** | **Include** | **Include** | **Include** | **Include** | **Exclude** | **Include** | **Include** | **Include** | **Include** | **Include** | **Include** | **Include** | **Unclear** |  |  |  |  |
| **Reviewer 2** |  |  |  |  |  |  |  |  |  |  |  |  |  |  |  |  |  |  |
| Were patient’s demographic characteristics clearly described? | Yes | Unclear | No | Unclear | Unclear | Unclear | Unclear | Unclear | Yes | Unclear | Unclear | No | Unclear | No | No | No | No | No |
| Was the patient’s history clearly described and presented as a timeline? | Yes | Yes | Yes | Yes | Yes | No | Yes | Yes | Yes | Unclear | No | Yes | No | Unclear | No | Yes | No | Yes |
| Was the current clinical condition of the patient on presentation clearly described? | Yes | Yes | No | Yes | No | No | No | No | Yes | No | No | No | No | Unclear | No | Yes | No | Yes |
| Were diagnostic tests or assessment methods and the results clearly described? | Yes | Yes | Yes | Yes | Yes | No | Yes | Yes | Unclear | Yes | Yes | Yes | Yes | Yes | Yes | Yes | No | Yes |
| Was the intervention(s) or treatment procedure(s) clearly described? | Yes | Yes | Yes | Yes | Yes | Yes | Yes | Yes | Yes | Yes | Yes | Yes | Yes | Yes | Unclear | No | No | Yes |
| Was the post-intervention clinical condition clearly described? | Unclear | No | Yes | Yes | No | No | Unclear | No | No | No | Unclear | Unclear | Unclear | No | Unclear | No | No | Yes |
| Were adverse events (harms) or unanticipated events identified and described? | No | No | No | No | No | No | No | No | No | No | No | No | No | No | No | No | No | No |
| Does the case report provide takeaway lessons? | Yes | Yes | Yes | Yes | Yes | Yes | Yes | Yes | No | Yes | Yes | Yes | Yes | Unclear | Yes | No | Yes | Yes |
| **Overall appraisal** | **Include** | **Include** | **Include** | **Include** | **Include** | **Exclude** | **Include** | **Include** | **Exclude** | **Include** | **Include** | **Include** | **Include** | **Include** | **Exclude** | **Exclude** | **Exclude** | **Include** |
| **Reviewer 3** |  |  |  |  |  |  |  |  |  |  |  |  |  |  |  |  |  |  |
| Were patient’s demographic characteristics clearly described? |  |  |  |  |  |  |  |  | Yes |  |  |  |  |  | No | No | No | No |
| Was the patient’s history clearly described and presented as a timeline? |  |  |  |  |  |  |  |  | Yes |  |  |  |  |  | No | Yes | No | Yes |
| Was the current clinical condition of the patient on presentation clearly described? |  |  |  |  |  |  |  |  | Yes |  |  |  |  |  | No | Yes | No | Yes |
| Were diagnostic tests or assessment methods and the results clearly described? |  |  |  |  |  |  |  |  | Unclear |  |  |  |  |  | Yes | Yes | No | Yes |
| Was the intervention(s) or treatment procedure(s) clearly described? |  |  |  |  |  |  |  |  | Yes |  |  |  |  |  | Unclear | No | No | Yes |
| Was the post-intervention clinical condition clearly described? |  |  |  |  |  |  |  |  | Unclear |  |  |  |  |  | Unclear | No | No | Yes |
| Were adverse events (harms) or unanticipated events identified and described? |  |  |  |  |  |  |  |  | No |  |  |  |  |  | No | No | No | No |
| Does the case report provide takeaway lessons? |  |  |  |  |  |  |  |  | Unclear |  |  |  |  |  | Yes | No | Yes | Yes |
| **Overall appraisal** |  |  |  |  |  |  |  |  | **Exclude** |  |  |  |  |  | **Exclude** | **Exclude** | **Exclude** | **Include** |

**Supplementary Table 4** - Summary of articles

| **Ref** | **Study Type** | **Size** | **Question** | **Study Criteria** | **Key Findings and Conclusions** |
| --- | --- | --- | --- | --- | --- |
| Wang et al [30] | Prospective  *Additional retrospective data* | 37  + 41^*^ | Prevalence of PSMA- FDG+ disease in high-risk early CRPCa post-RP with negative conventional imaging | - Rising PSA ≤ 2ng/ml  - Testosterone < 50 ng/dL  - PSA doubling time ≤ 10 months  - N0M0 on CT and bone scan | Subsequent addition of FDG after PSMA PET/CT increased detection of N+/M+ from 65% to 73%  **Additional retrospective data:* PSMA- FDG+ disease found in 33% of CRPCa, 6% of HSPCa |
| Chen et al [33] | Retrospective | 72 | Prevalence of FDG-avid disease in BCR with negative PSMA PET/CT | - PSA > 0.2 ng/ml post-RP  - Not on androgen-deprivation  - N0M0 on Ga68 PSMA PET/CT | FDG-avid disease found in 17% of patients  90% positive FDG PET/CT with high potential criteria (PSA ≥ 2.3 ng/ml and Gleason score ≥ 8) versus 0% in low potential (PSA <2.3, Gleason score <8) |
| Shi et al [34] | Retrospective | 138 | Ability of dual PSMA/FDG PET/CT to differentiate peripheral ganglia from lymph node metastases | - FDG and PSMA PET/CT performed within 2 weeks  - Any indication | Significantly less ganglia showed showed FDG uptake (p<0.001)  PSMA/FDG PET detected N+ when both avid (PSMA SUV_max_ >2.05, FDG SUV_max_ >4.1; n=43/47, 92%) compared to low or no avidity (PSMA SUV_max_ <2.05, FDG SUV_max_ <4.1; n=3/334, 1%). |
| Zhou et al [35] | Retrospective | 21 | Intra-individual comparison of dual FDG and PSMA PET/CT in primary staging | - Pathological diagnosis of PCa  - No prior treatment | 100% had local PSMA avidity in prostate versus 66.7% with local FDG uptake  PSMA PET/CT identified more bone (50 vs 32) and lymph node (25 v 22) metastases, with less benign lesions (21% vs 49%) than FDG PET |
